# 3D direct-write printing of water soluble micromoulds for high-resolution rapid prototyping

**DOI:** 10.1016/j.addma.2022.103019

**Published:** 2022-10

**Authors:** Saja Aabith, Richard Caulfield, Omid Akhlaghi, Anastasia Papadopoulou, Shervanthi Homer-Vanniasinkam, Manish K. Tiwari

**Affiliations:** a Nanoengineered Systems Laboratory, UCL Mechanical Engineering, University College London, London WC1E 7JE, UK; b Wellcome/EPSRC Centre for Interventional and Surgical Sciences, University College London, London W1W 7TS, UK; c UCL Department of Medical Physics and Biomedical Engineering, University College London, London WC1E 6BT, UK; d Leeds Vascular Institute, Leeds General Infirmary, Great George Street, Leeds LS1 3EX, UK

**Keywords:** High-resolution 3D printing, Micromoulding, Water based ink, Precision prototyping, Flexible/soft material prototyping

## Abstract

Direct-write printing has contributed tremendously to additive manufacturing; in particular extrusion based printing where it has extended the range of materials for 3D printing and thus enabled use across many more sectors. The printing inks for direct-write printing however, need careful synthesis and invariably undergo extensive preparation before being able to print. Hence, new ink synthesis efforts are required every time a new material is to be printed; this is particularly challenging for low storage modulus (G’) materials like silicones, especially at higher resolutions (under 10 µm). Here we report the development of a precise (< 10 µm) 3D printable polymer, with which we 3D print micromoulds which are filled with standard silicones like polydimethylsiloxane (PDMS) and left to cure at room temperature. The proof of concept is demonstrated using a simple water soluble polymer as the mould material. The approach enables micrometre scale silicone structures to be prototyped with ease, away from the cleanroom.

## 1 Introduction

The manufacturing world has taken big strides with the advent of additive manufacturing (AM) and 3D printing. There has been a surge of AM techniques that have been introduced within the last two decades alone, in particular, direct-write techniques [1] for small-scale manufacturing (<10 µm) [2]. Amongst these, the direct-write assembly approach pioneered by the Lewis group [3] in combination with the relevant material science exploitation of printable ink formulations [4] brought about excellent manufacturing control in various sectors including photonics [5], [6], microfluidics [7], biomaterials [8], etc. [9] without requiring lithographic masks or similar expensive procedures [4]. However, each material to be deposited/printed with, needs much fine tuning of the ink properties such as viscosity [10], viscoelasticity [11], and evaporation rate [12] in order to be 3D printable and form self-standing structures. Additionally, at high resolution the ink would need to undergo further adjustments based on the surface/sample onto which it would be printed. This is due to the wide-ranging surface energies that are associated with different types of surfaces, which play a critical part in ensuring appropriate spreading and good adhesion of the ink while printing [13]. The many variables involved in ink optimisation make the process extensive and time consuming. This is further complicated for inks where the curing is unattainable without external interference of temperature, radiation, etc. Such inks can be tuned to get 2D printing, but extending this to achieve 3D printability is challenging since the consecutively printed layer relies on the previously printed layer to be cured in time. Silicones, fall under this umbrella. The capability to manufacture precise silicone structures effectively has been much sought after due to the wider use of silicones that spans from electrical insulations in microelectronics to medical grade implants, contact lenses and catheters in the medical world. This is due to silicone's valuable properties including high heat resistance and low surface energy [14], good flexibility [15], low toxicity, etc. [16]. The range of applications could be further expanded with increased precision and control in sub-millimetre scale silicone fabrication [17], assuredly leading to silicone materials playing an unparalleled role in many important areas such as nanotechnology.

Yirmibesoglu et al. [18] developed an extrusion system that incorporates an active mixer [19] and a controlled heat treatment [20] for successfully 3D printing 2-part (base & curing agent) silicone material. However, their system relies on a convective heater fan temperature of 80 °C and a heated bed temperature of 50 °C for the successful printing and curing of the two-part silicone. The temperature dependence of the system limits its use and makes printing on temperature sensitive surfaces impossible. Additionally, the resolution of the printed structures is limited to the nozzle size of 1.3 mm.

Eggbeer et al. [21] evaluated direct and indirect AM for the fabrication of maxillofacial prostheses. The direct AM process involved 3D printing the body of the prosthesis with a soft acrylate based material and wrapping it with a thin layer of silicone. The indirect AM procedure consisted of 3D printing a mould for the nasal prosthesis and filling it with silicone. The mould made (indirect AM) prosthesis was found to be clinically suitable and even rated to be better than conventionally fabricated prostheses. Similar 3D printing techniques like fuse deposition modelling (FDM), etc. have been used for 3D printing moulds for prosthetics [22], but they lack in printing resolution and ease in the demoulding process, especially for sub-millimetre scale moulding (micromoulding) [23]. Additionally, for the case of complex structures the moulds need to be fractured (potentially damaging the moulding) or even bleached away as demonstrated by Jung et al. [24] who 3D printed polycaprolactone (PCL) - gelatine scaffolds as moulds for cell culture studies with an alkaline soluble photopolymer resin and projection-based microstereolithography (pMSTL). However, the moulds had to be washed away with alkaline solution (0.5 M NaOH), which has a pH of 13.69, that is comparable to the pH of bleach, thus limiting the application range due to product safety considerations. Similarly, Therriault et al. [25] reported a similar indirect AM approach for fabricating microvascular networks. However, the print resolution was limited to the nozzle size of 200 µm. Additionally, the printed scaffold required high temperatures (75 °C) to get removed. More recently, Kim et al. [26] have reported a magnetic field assisted direct-write printing approach of a ferromagnetic loaded elastomer matrix. However, the approach is limited to a nozzle size of 200 µm and the printed scaffold – made up of silicone catalyst and silica nanoparticle composite – requires an organic solvent like chloroform to dissolve it away.

Multiple groups [27], [28], [29] have demonstrated micrometre mould printing us stereolithography (SLA) and digital light processing (DLP) techniques. However, in particular for mould printing, both approaches inherently suffer from challenges that the direct-write approach overcomes. SLA and DLP printing are relatively slow (often limited by slow photopolymerisation rates) [30], expensive (due to the photosensitive resin and the need of a laser source) [31] and especially for mould printing, they requires careful tuning of printing angles, in order to achieve the highest resolution [27]. This can lead to the need for printing multiple moulds to achieve desirable precision. Inkjet printing limited by the need for careful rheology control to ensure droplet/filament break-up and particularly challenging for non-linear, viscoelastic inks such as those considered here.

Here, in order to overcome the above mentioned limitations of the direct-write approach, we report the development of a 3D printable water-based polymer ink which is 3D printed to give hollow structures – micromoulds - that are then filled with the target material, like silicones such as Polydimethylsiloxane (PDMS) or Ecoflex. The water-based ink is clearly safe and is introduced to emphasise the simplicity of the approach. The filled mould were cured at the appropriate temperature relevant to the filler material and then simply washed away with few water droplets, leaving behind the cured moulding (*see* Fig. 2). This approach decouples the extensive ink synthesis – the major challenge of the direct-write approach [32] – from 3D direct-write printability, thus enabling simplistic advanced control in micrometre scale silica 1 AM without any harmful solvents, all while outside a cleanroom. This arrangement paves the way for high level integration in many sectors including the healthcare sector. This is of particular relevance to sectors which require manufacturing/printing of specific silicone structures onto pre-existing platforms, without causing any damage to the underlying device, which may be expensive and/or delicate.

## 2 Materials and methods

### 2.1 Inks

All the inks that were used are known to be safe and biocompatible and were prepared outside the cleanroom. Polyvinylpyrrolidone (PVP) was purchased from Sigma Aldrich. The ink was prepared by dissolving PVP mol. wt. 360 kDa in deionised (DI) water via stirring at 60 rpm at room temperature over 6 days. The relatively mild stirring process nudges the linear PVP strands to organise themselves into bundles, which are held together by the water through hydrogen bonding to C=O on the pyrrolidone ring. This inter-strand hydrogen bonding is enable by the free rotation of the pyrrolidone ring on the C—N bond [33]. The homogeneous solution so obtained is printable directly, with suitably chosen nozzle diameters, without any further control on chemistry. The PVP ink was prepared in varying concentrations from 10 to 40 wt% in order to create a suitable range for testing 3D printability. Here we define 3D printability as the ability to form stable printed structures. Two different silicones were used to fill the printed moulds. Firstly, polydimethylsiloxane (PDMS, Sylgard 184) was purchased from Fisher Scientific and prepared in the recommended ratio of 10 parts of silicone base to 1 part of curing agent (10:1). This was stirred for 3 min and then desiccated in a vacuum chamber for another 20 min in order to remove the trapped air bubbles inside the silicone mixture. Additionally, Ecoflex (Ecoflex series 00–10–00–50) was purchased from Bentley Advanced Materials. Ecoflex was prepared in the recommended ratio of 1 part A to 1 part B (1:1). This was stirred for 2 min and desiccated in the vacuum chamber for 10 min.

### 2.2 Rheology of PVP inks

The effect of the different PVP concentrations on the viscosity and viscoelasticity of the printing inks was rheologically studied using a rheometer (DHR-3, TA instruments). All tests were carried out at room temperature (controlled), with a sandblasted parallel plate geometry (40 mm) and a sample gap of 500 µm. The sandblasted parallel plate geometry was used to avoid any apparent wall slip [34]. The inks were subjected to a flow ramp (steady-state response) and oscillatory amplitude sweep. The amplitude sweep was performed at a frequency of 1 Hz over a torque range of 0.1–10,000 μN m at 5 points per decade.

### 2.3 Printing

**Fig. 1:**
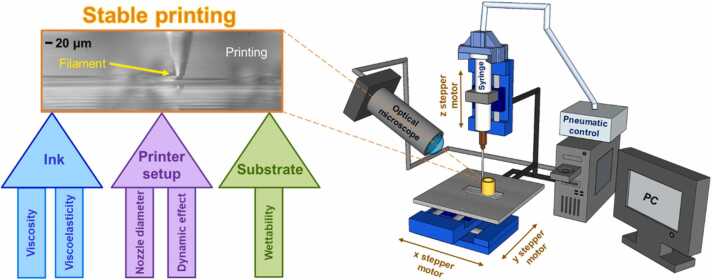
Schematic of the direct-write assembly approach printing setup and printing variables influencing resolution and print quality.

The direct-write printing system essentially consists of four main components: the nozzle assembly, the stage controllers (PC controlled) that control the three micro-translation stages (x, y and z; each with a motion step size of 50 nm and repeatability of 100 nm), an optical microscope focusing on the aperture of the nozzle and a nitrogen gas supply in combination with an electronic pneumatic regulator for applying set pressures to the syringe barrel to force the ink out of the nozzle. The nozzle assembly comprises a syringe barrel that is filled with the required ink to be printed, a piston and an attached glass nozzle, which can be thought of as the ‘pen’ of the setup. The glass nozzles were fabricated using a micropipette puller (Sutter instrument, P-1000) providing varied apertures that can be as low as 600 nm. The nozzle aperture was determined based on the target application/resolution. The micro-translation stages (Physik Instrumente, M-111.12 S) have a step resolution of 50 nm, a working range of 15 mm and a max velocity of 1 mm/s. The three stepper motor controllers (Physik Instrumente, model: C-663) were controlled via software (Physik Instrumente, PIMikroMove) and our LabVIEW (National Instruments) script. It should be noted that another setup with a higher step resolution of 1 nm was also used instead of the micro-translation stages. This was a piezoelectric actuated nanopositioning stage (Physik Instrumente, P-615.3CL Nanocube) with a step resolution of 1 nm and a working range of 350 µm x 350 µm x 250 µm. The higher step resolution of the piezoelectric stage ensured smoother curvatures when printing curved moulds. The print quality and resolution were primarily controlled by carefully tuning the rheological properties (viscosity and viscoelasticity) of the inks to be printed with, the printing speed of the micro-translation stages and the surface wetting behaviour of the sample to be printed on top of (*see* Fig. 1). The substrate wettability was not characterised specifically because we only used high-energy (wettable) substrates such as glass, copper, aluminium and stainless steel to ensure good adhesion of printed structures with the substrate. A proper investigation of the role of substrate wettability is beyond the scope of the current work and indeed will require a focussed investigation. However, substrates were cleaned in acetone and isopropanol to remove any contaminations. No attempt was made to alter surface through surface roughening or changing surface energy (wettability) through functionalisation or plasma treatment. The addition of polyvinylpyrrolidone to water (i.e. our inks) should have lower surface tension [35] and should facilitate better wettability/adhesion.

### 2.4 Micromould printing – decoupling ink synthesis and printability from rapid prototyping

**Fig. 2:**
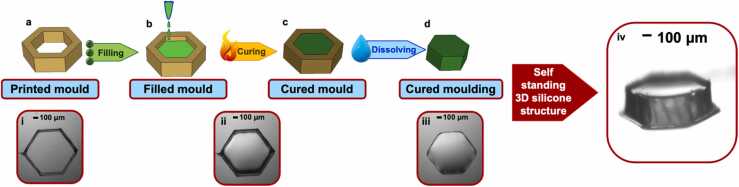
Mould process: a.) Printing PVP mould b.) Filling printed mould with PDMS via nozzle c.) Curing mould with PDMS content at RT for 48 h or in an oven at 125 °C for 20 min d.) Pipetting few water droplets onto the mould in order to dissolve PVP moulding and leaving behind cured and structured PDMS i.) Printed hexagonal structure ii.) PDMS filled and cured mould iii.) top view of cured moulding without mould iv.) 3D view of cured moulding without mould.

For the mould fabrication, hollow 3D structures were printed with our developed PVP inks at a speed of 0.2 mm/s and nozzle apertures ranging from 5 to 35 µm (*see* Fig. 2*a* and Supplementary Video 1). The printed structures were then filled with PDMS, Ecoflex or composites like PDMS with carbon black nanoparticles. This was done by loading a syringe barrel with the desiccated silicone, attaching a 1–5 µm nozzle to the syringe, lowering and aligning the nozzle assembly above the printed mould, the operator applying sufficient pressure (0.1–0.3 bar) to the piston to just force the filler ink out of the nozzle and retracting the nozzle assembly once the mould was filled to the brim (*see* Fig. 2*b*). The mould was considered to be ‘filled to the brim’ when the inner edge of the mould was not visible. All steps of which were completely observable via the optical microscope that was tilted at 45° (*see* Fig. S1 and Supplementary Video 2). The filled mould was left to cure in an oven at 125 °C for 20 min or at room temperature for 48 h, such that the PDMS cured completely (*see* Fig. 2*c*). The mould with the cured moulding was placed outside the oven at RT for another 10 min to cool down completely. The water soluble PVP mould was then easily washed away with a few water droplets by simply pipetting water droplets onto the mould, resulting in the moulding (PDMS structure) to remain behind (*see* Fig. 2*d*). This was done for different structures including 3D hexagons (*see* Fig. 2*i-iv*).

Supplementary material related to this article can be found online at doi:10.1016/j.addma.2022.103019.

The following is the Supplementary material related to this article Video 1, Video 2.

Video 1

Video 2.

## 3 Results and discussion

### 3.1 Rheology of PVP inks

**Fig. 3:**
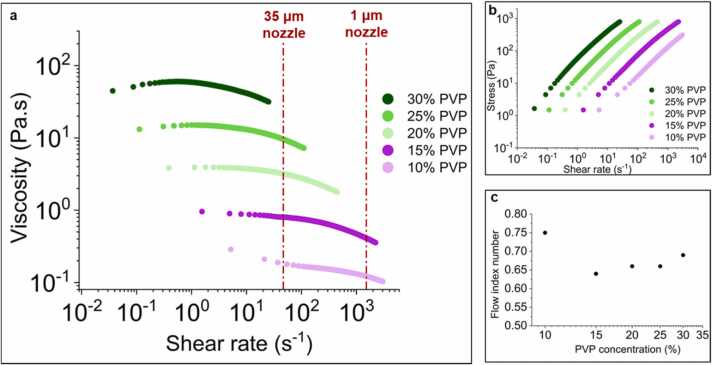
Steady-state response of PVP inks: a.) Viscosity as a function of shear rate b.) Oscillation stress as a function of shear rate c.) Flow index number for PVP ink concentrations.

The flow behaviour and viscoelastic properties for 10, 15, 20, 25 and 30 wt% PVP ink concentrations were obtained. From the steady-state response (*see* Fig. 3*a*), the zero shear viscosity values increase from 0.3 Pa.s to 40 Pa.s with increase in PVP concentration. The inks show clear shear thinning across the PVP concentrations in the shear rate ($\dot{\gamma }$) region of 46 – 1600 s^-1^, which corresponds to our printing speed of 0.2 mm/s for nozzle sizes 1–35 µm; calculated using ${\dot{\gamma }}_{\max}=4\dot{Q}/\pi r^{3},$ where r is the nozzle radius and $\dot{Q}$ is the volume flow rate, which is calculated as $\dot{Q}=V_{p}\pi r^{2}$ with $V_{p}$ being the printing speed [36]. The higher concentration inks (20–30 wt%) show small shear thickening for the lower shear rates, which can be attributed to the intermolecular entanglements which can form during low shear rates [37]. These entanglements will get destroyed during the higher shear rates, leading to the reduced viscosity (shear thinning). The developed inks showed no thixotropic behaviour. This was determined by looking for hysteresis in another flow ramp test (ramp up and down), which was applied on 10 wt% and 25 wt% inks. Viscosity as a function of shear rate was shown (*see* Fig. S2), where the ramp up and down data points overlapped, suggesting no hysteresis behaviour. The non-thixotropic behaviour of the inks was further ascertained by a 3 step test (*see* Fig. S3), where a low shear rate of 1 s^-1^ was applied for 10 s, followed by applying an increased shear rate of 100 s^-1^ for 10 s and then in the third step reducing the shear rate to 1 s^-1^ for another 10 s. There was no time delay visible and the viscosity followed the shear rate instantaneously.

The flow index numbers (n) for the different PVP concentrations (*see* Fig. 3*c*) were obtained from least square fitting a power law model $\tau =\alpha {\dot{\gamma }}^{n}$ to the shear stress (τ) as a function of shear rate ($\dot{\gamma }$), where α is the slope (*see* Fig. 3*b*). The flow index numbers for the different PVP concentrations are very similar, ranging from 0.64 to 0.75. These similar flow index values suggest similar flow behaviour across all of the different PVP concentrations tested.

**Fig. 4:**
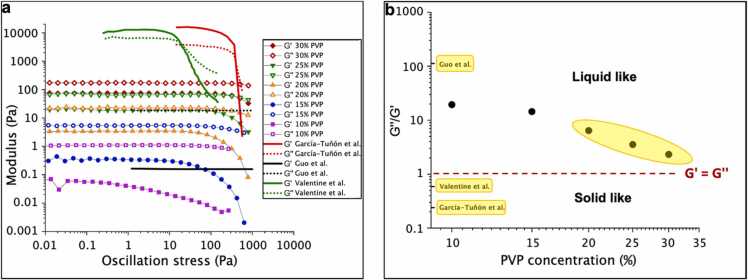
Viscoelastic properties of PVP inks: a.) G’ and G’’ as a function of oscillation stress for the different PVP concentrations and other reported inks [38], [39], [40] b.) Ratio of loss modulus to storage modulus as a function of PVP concentration in comparison to other inks [38], [39], [40], with the 3D printable inks highlighted in yellow.

The viscoelastic response (*see* Fig. 4*a*) shows that the storage modulus is lower than the loss modulus for all PVP concentrations (G’<G’’). This agrees with the findings of Guo et al. who used UV assisted 3D printable polyimide ink [38]. However, it is in contrast to the 3D printable graphene oxide (GO) based inks from García-Tuñón et al. [39] and the 3D printable silver flake loaded thermoplastic polyurethane (AgTPU) from Valentine et al. [40], both of which have a higher storage modulus than loss modulus (G’>G’’). Determining the dominant modulus is important as this feature has an influence on the ink consistency and print resolution (*see* Fig. 4*b*): the GO based ink and the AgTPU ink are more paste like and are printed with 510 µm and 200 µm nozzles respectively, which is bigger than the 90 µm nozzles used for the polyimide ink, as it was more liquid-like and also needed UV assistance while printing to help the curing/printing process. It should be noted that the biggest nozzle size that was used in our work was 35 µm, without any UV cure assistance.

As for the inks that have been developed in this work, despite G” being greater than G’, stable structures were successfully printed thanks to the evaporation of the solvent (water). The evaporation became stronger with reduction in nozzle size, such that the evaporation time τ_v_ ~ d^2^, where d is the filament diameter. However, it should be also noted that evaporation also has a negative impact of causing nozzle clogging, particularly for finer nozzles.

The G’’/G’ plot in Fig. 4b shows the expected outcome of the liquid-like inks approaching the gelation point (G’ = G’’) [41] when increasing the polymer content of the ink. This is due to the increased formation of polymer networks when the polymer content is increased.

### 3.2 Printability of PVP inks

In order to develop a 3D printable water based PVP ink, the PVP concentration was varied from 10 to 40 wt% and tested with different nozzle (tip) apertures ranging from 1 µm to 35 µm. The 40 wt% PVP ink was unprintable and always led to nozzle clogging. By ‘nozzle clogging’ we mean that the ink stops flowing through the nozzle due to one of two reasons. The first reason applies for the 40 or more wt% PVP inks only, where the ink was too viscous so that it could not flow through the nozzle. The second reason was due to the opening (aperture) of the nozzle getting slowly blocked by PVP ink that accumulated over the printing duration, which mostly occurred for the smaller (<5 µm) nozzles. This indeed also highlights the challenges faced in direct-write printing at such resolutions.

**Fig. 5:**
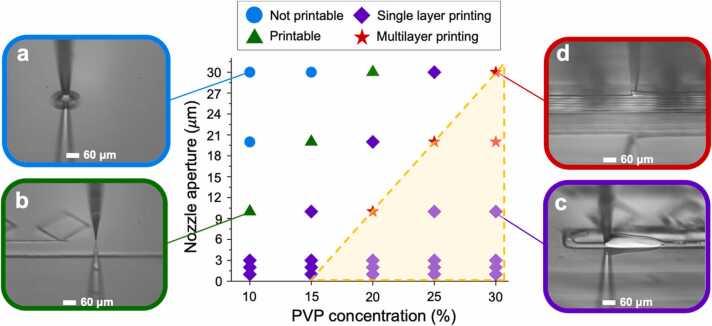
Map of printability of the inks with different PVP concentrations with different nozzle sizes. Four regions are identified: a.) ‘Not printable’ meaning that the ink would just wet the substrate without forming a surface adhering filament b.) ‘Printable’ meaning that the ink facilitated filament formation that adhered to the surface, but unable to complete the initial layer due to either nozzle fracture, clogging or overflowing c.) ‘Single layer printing’ meaning printing could be completed for a single layer, but unable to proceed to multiple layers due to nozzle clogging or fracture d.) ‘Multilayer printing’ meaning that the ink was 3D printable and enabled the formation of stable/self-standing structures without the nozzle clogging or fracturing at consecutive layers. Shaded region represents the multilayer printing region if nozzle stability was not a factor.

Fig. 5 summarises the printability of the different PVP concentration inks with respect to nozzle sizes. It should be noted that the micropipette puller machine fabricated varying nozzle sizes, which resulted in the implementation of a nozzle size bin classification. However the data points (1–3, 10, 20 and 30 µm) in Fig. 5 were the actual sizes of the nozzles that were frequently printed with (>5 print runs each). The rest of the nozzle sizes within each classification were test printed with fewer times than the data point nozzle sizes.

With nozzle sizes of 35–15 µm and a PVP concentration of 30 wt%, ‘multilayer printing’ - the ink was 3D printable and able to form self-standing structures without the nozzle clogging or fracturing at consecutive layers - was achieved (*see* Fig. 5*d*). Being able to 3D print multiple layers is crucial for making fully formed 3D structures. ‘Multilayer printing’ was also achievable with nozzle sizes of 25–15 µm and PVP concentration of 25 wt%. Similarly, multilayer printing was attained with nozzle sizes of 15–5 µm and the 20 wt% PVP ink.

It should be noted that the extruded filament did show die swelling effect after exiting the nozzle [42]. Additionally filament wetting may reduce the final dried up height of the extruded filament on the substrate. The amount of wetting was different for different substrates (different surface energies), but based on our print runs, the wetting on glass substrate causes the filament width to be ~1.5 × the nozzle aperture. So that for a 10 µm nozzle, the extruded filament would be ~15 µm wide and ~10 µm high on the substrate. These dimensions were highly reproducible for given ink PVP concentration and nozzle size. This enabled us to build vertical structures while keeping the vertical increment for each layer constant. So that for printing a 100 µm high structure with a 10 µm nozzle, the stages are programmed such that they increment 10 µm after each layer for 10 layers. The approach was successfully exploited to build multi-layered structures without the nozzle losing fluid contact in one of the consecutive print layers (*see* Fig. S4). For all PVP concentrations, using nozzles under 5 µm only enabled ‘single layer printing’. Single layer printing meaning being able to print a single complete layer, after which the nozzle either clogs up or breaks (*see* Fig. 5*c*).

The 10 wt% PVP ink with nozzle sizes of 35–15 µm was not printable. In this case, the ink simply wet the surface without forming a filament (*see* Fig. 5*a*). Nozzle sizes less than 14 µm but bigger than 3 µm were ‘printable’ with the 10 wt% PVP ink - being able to form filaments that are adhering to the surface, but not being able to complete the first layer due to either nozzle fracturing, clogging up or the ink overflowing (*see* Fig. 5*b*).

As shown in Fig. 4 all of our inks are liquid-like and approaching the gelation point with increase in polymer content. However, among the printable inks, from the least liquid like 30 wt% PVP ink to the most liquid-like 10 wt% PVP ink we developed, we could not achieve multilayer printing with nozzles sizes that were smaller than 5 µm, which broke frequently. This suggests that despite having dominating G’’ and sufficient G’ to form filaments, the stability of the glass nozzles is more of a dominating factor to achieve multilayer printability, since a low G’ is sufficient to break the fine glass nozzles and hinder continuous printability to achieve multilayer printing. If the glass nozzles under 5 µm were made with a material of sufficiently high strength, then multilayer printing could be achievable for all the nozzle apertures and their respective ink concentrations in the shaded region in Fig. 5. However, in order to verify this another study that uses other glass tips that have sufficiently higher Young’s modulus to overcome the stress applied by the inks should be conducted. Yuk and Zhao [43] developed a quantitative phase diagram to map different printing modes for viscoelastic inks (*see* Fig. S5). The phase diagram maps different printing modes of viscoelastic inks with the help of non-dimensional parameters (H*, V*), where H* =H/αD and V* =V/C, with H being the gap between the tip and the substrate, α being the die-swelling ratio, D being the diameter of the tip, V being the tip moving speed and C being the extrusion rate. Our developed water based PVP inks all die-swell upon extrusion (V*≥1) and H* =0.41 (H=0.5 *D, α = 1.23, see Fig. S5a). The phase diagram doesn’t cater for H* <1. Separately, the phase diagram doesn’t take into consideration mechanical failure of the nozzles, since the group uses metal tips that have apertures of ≥ 50 µm and inks that are solid-like (G’’/G’ < 1). But in our work we are working with much smaller nozzle apertures (1–35 µm) that are made of fragile glass capillaries that can break due to ink accumulation or clogging and our inks are liquid-like (G’’/G’ > 1). This is the reason why our plot is more suitable for our printing conditions, where mechanical failure of glass nozzles due to ink accumulation or clogging must be taken into account, since mechanical failure hinders printability.

### 3.3 Moulding and prototyping

The micromoulds were successfully printed onto various substrates including glass, copper, aluminium and stainless steel, owing to the good adhesion properties of PVP. The printable substrate range extends the potential applications of this moulding approach further.

**Fig. 6:**
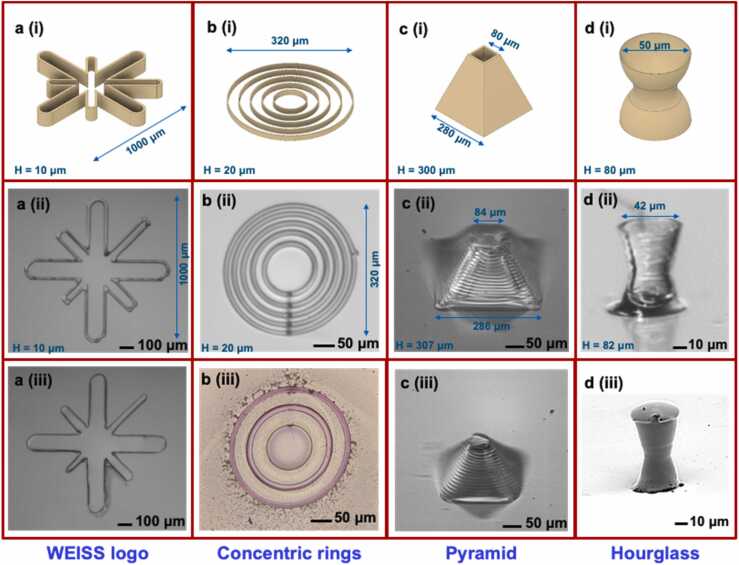
Printed moulds and their mouldings: a.) (i) WEISS (Wellcome/EPSRC Centre for Interventional and Surgical Sciences) logo mould schematic, (ii) printed mould and (iii) PDMS moulding b.) (i) Concentric rings mould schematic, (ii) printed mould and (iii) moulding of PDMS with embedded gold nanoparticles c.) (i) Pyramidal structure mould schematic, (ii) printed mould and (iii) moulding of Ecoflex d.) (i) Hourglass mould schematic, (ii) printed mould and (iii) moulding of PDMS.

Some of the moulds that were printed and their corresponding mouldings are shown in Fig. 6. The star shape logo of UCLs’ Wellcome/EPSRC centre for Interventional and Surgical Sciences (WEISS) was printed on glass with a 10 µm nozzle and 25 wt% PVP ink. The WEISS logo was printed at a velocity of 0.2 mm/s, so that the whole printout took 37 s. The printed logo was consecutively filled with PDMS 10:1 (*see* Fig. 6*a*). The printed mould was only 1 layer high (~10 µm), thus showing how suitable a water-based mould is in getting filled with a hydrophobic filling (PDMS). The hydrophobicity of the PDMS stops it from crossing the printed mould boundaries.

The concentric rings were printed with a 10 µm nozzle and 20 wt% PVP ink to create a multi-layer design of height 20 µm (*see* Fig. 6*b(i, ii)*). The gaps of the concentric rings were filled with PDMS in an alternating manner, i.e. the first filling would be at gap 1 (centre), then gap 3 and then finally gap 5. This was to demonstrate the micrometre precision control in the filling process. Then the PDMS was cured as before. The supporting mould was then washed away, leaving behind 3 concentric PDMS rings. Following the procedure outlined by Goyal et al. [44] the PDMS rings were then submerged in a gold salt solution for 24 h. The platinum curing agent in the Sylgard 184 PDMS catalyses the reduction of the gold salt in situ to give gold nanoparticles, which embed themselves into the PDMS structure (*see* Fig. 6*b(iii)*). This is a facile method of synthesising gold nanoparticle - PDMS composites, such composite materials have uses in optical and photoacoustic sensing applications [45]. Here it serves as a means to demonstrate the versatility of our approach to create reactive components leading in situ nanoparticle formation, thereby facilitating soft nanocomposite formation.”

Similarly, more complex 3D structures like pyramids and hourglasses were printed. A pyramidal mould was printed with a 15 µm nozzle and 30 wt% PVP ink (*see* Fig. 6*c(i, ii)*). The Ecoflex moulding is of good quality, where the number of layers can be seen from the moulding itself. The tip of the pyramid is under 50 µm in diameter (*see* Fig. 6*c(iii)*). Additionally, an hourglass shaped mould was printed with a 10 µm nozzle and 20 wt% PVP ink with the higher resolution printing setup at a reduced speed of 0.04 mm/s to avoid stringing on the curved structure. The complete hourglass printout took 4 min and 24 s. The mould was filled with PDMS, then cured and demoulded as before. The hourglass mould (*see* Fig. 6*d(ii)*), is a smaller mould than the other moulds (Fig. 6a-c(ii)), and thus minor defects, such as the small strand that is formed when retracting the nozzle after the mould printing can affect the quality of the moulding. However, the extra string of PVP that was formed while retracting the nozzle after printing (*see* Fig. 6*d(ii)*), was washed away in the demoulding process (*see* Fig. 6*d(iii)*).

Following this procedure, we highlight how silicone microstructures manufactured using our technique can be utilised to create functional nanocomposite materials across many different areas, either directly or as an initial step in a more complex manufacturing process. It should be noted that the overall manufacturing time of our approach is only a fraction of the manufacturing time of other high-resolution manufacturing approaches, that are predominantly subtractive approaches. To achieve 10 µm repeatability in a typical photolithographic process would comprise of multiple challenging steps to obtain just a soft lithographic pattern, which could take almost 2 days. However, our approach achieves this within minutes.

Still, there are some limitations that need to be overcome in order to enhance our approach even further. Moulding a scaffold like a truss structure or a woodpile structure would be challenging with our approach. However, soft lithography could serve as a potential workaround for some challenging structures like the woodpile structure, where the negative of the mould – closely spaced cubes with a surrounding wall - would be printed with PVP and moulded with PDMS. This way a PDMS master is obtained (PDMS soft mould). This can be vapour treated with trichlorosilane for easy demoulding [46]. The treated PDMS soft mould can be used to mould a PDMS woodpile structure. Additionally, the printed moulds leave behind sub-micron sized indents of the layers on the moulding, which can be a limitations for certain applications like microlenses.

To place our work in context, Fig. 6*.* clearly shows the potential of our approach to prototype otherwise difficult to structure materials like PDMS. Although, a thorough investigation of the printing fidelity and precise obtainable resolutions are beyond the scope of the current work, some general inferences can be drawn. Thus, the printing fidelity depends on three different features:

(i) accuracy of the positioning system used
(ii) the difference between the nozzle size and the extruded filament diameter (which depends on the die-swell ratio and the substrate/inter-layer wettabalities of printed filament), and
(iii) the mechanical stability of the printed structure.

As mentioned above, the positioning system that we have employed has the coarsest repeatability of 100 nm; we also used a system with sub-nm position repeatability. Therefore this is likely to have a small effect on the accuracy.

As mentioned previously, we found that the extruded filament height was the same as the nozzle diameter and the extruded filament width was consistently ~1.5 x the nozzle diameter. Lastly, we observed no changes in the diameter of the printed PVP filaments and the shrinkage of the PDMS is known to be ca. 1% [47]. There are noticeable differences in the dimensions between the design and the final prototype. For example, in the hourglass case, the design diameter of the top of the hourglass was 50 µm and the corresponding diameter on the mould was 42 µm. This is due to material (ink) overflow. Note that this overflow is very limited when the aspect ratio of the printed structure is small. Overall, this leads to ca. 2% lower dimensions for the first few layers of printing (compared to the design dimension) and moves to ca. 40% for the highest aspect ratio such as the pyramid (*see* Fig. 6*c*) and hourglass (*see* Fig. 6*d*).

It is also important to note that with our PVP based moulds it is not possible to remove the filament traces after printing. However, we can reduce the imprint size by using a translating stage with finer step precision, just as we did for the hourglass in Fig. 6*d*, where the imprints are hardly visible. Melt processable polymers may possibly allow a complete smoothing of the imprints. However, they are beyond the scope of the current work and PDMS is known to faithfully replicate geometries within a precision of a few nm [48].

## 4 Conclusion

To facilitate precision prototyping of silicones as an exemplar material, using high-resolution 3D direct-writing, we developed and rheologically characterised a set of water soluble PVP inks. These PVP inks facilitated rapid high-resolution prototyping, enabling micrometre structures to be fabricated with ease outside of any cleanroom environment which is typically required for such high-resolution manufacture (e.g. for mould manufacture). A number of exemplar micromould geometries were printed using this approach and silicone and silicone nanocomposite structures were successfully demonstrated. The study and the approach introduces a safe and simple approach to prototype high-resolution (<10 µm) structures using direct-write 3D printing, without the need for exhaustive formulation and optimisation of inks. This tends to be a major challenge because often the material with suitable properties for a desirable application lacks the features to be 3D printed. Although our demonstrations concentrate on silicones (that lacked the required viscoelastic features to be printed directly), the freedom gained from removing the need for painstaking ink optimisation processes will open the door to a variety of soft, 3D microstructures prototyping for wide range of applications in areas such as flexible electronics (i.e. sensors) and bioengineering (i.e. implants, etc.).

## CRediT authorship contribution statement

**Manish K. Tiwari:** Writing – review & editing, Writing – original draft, Supervision, Resources, Project administration, Methodology, Investigation, Funding acquisition, Conceptualization. **Omid Akhlaghi:** Validation, Methodology, Investigation, Formal analysis. **Richard Caulfield:** Writing – review & editing, Visualization, Methodology, Formal analysis. **Saja Aabith:** Writing – review & editing, Writing – original draft, Validation, Methodology, Investigation, Formal analysis, Data curation, Conceptualization. **Shervanthi Homer-Vanniasinkam:** Writing – review & editing, Supervision, Investigation, Conceptualization. **Anastasia Papadopoulou:** Writing – review & editing, Methodology, Investigation.

## Declaration of Competing Interest

The authors declare the following financial interests/personal relationships which may be considered as potential competing interests: Manish K. Tiwari reports financial support was provided by Wellcome EPSRC Centre for Interventional and Surgical Sciences. Manish K. Tiwari reports financial support was provided by European Research Council (ERC) under the European Union’s Horizon 2020 research and innovation programme under grant agreement no. 714712. Saja Aabith reports financial support was provided by EPSRC DTP studentship award.

## Data Availability

Data will be made available on request.
