# Supplementary material for: 3D direct-write printing of water soluble micromoulds for high-resolution rapid prototyping
Source: Addit Manuf. 2022 Oct;58:None. doi: 10.1016/j.addma.2022.103019 (PMC10499758; doi:10.1016/j.addma.2022.103019)
Supplement: Supplementary file 3 — Supplementary material [file mmc1.docx]

# Supplementary material

**3D direct-write printing of water soluble micromoulds for high-resolution rapid prototyping**

Saja Aabith^1,2^, Richard Caulfield^1,2,3^, Omid Akhlaghi^1,2^, Anastasia Papadopoulou^1,2^, Shervanthi Homer-Vanniasinkam^2,4^, Manish K. Tiwari^1,2^*

^1^Nanoengineered Systems Laboratory, UCL Mechanical Engineering, University College London, London, WC1E 7JE, U.K.

^2^Wellcome/EPSRC Centre for Interventional and Surgical Sciences, University College London, London, W1W 7TS, U.K.

^3^UCL Department of Medical Physics and Biomedical Engineering, University College London, London, WC1E 6BT, U.K.

^4^Leeds Vascular Institute, Leeds General Infirmary, Great George Street, Leeds LS1 3EX, UK

*Corresponding author: m.tiwari@ucl.ac.uk

**Mould filling**

*Figure S1: Mould filling: a.) Aligning and lowering nozzle (1 - 5 μm) into printed mould b-d.) Applying sufficient pressure (0.1 - 0.3 bar) to fill the mould with composite of PDMS with 0.1 wt% carbon black nanoparticles dispersed e.) Moving nozzle to the top of the printed mould f.) Removing the pressure and raising the nozzle out of the filled mould.*

**Non-thixotropic behaviour of developed inks**

In order to assess the shear thinning and time dependency behaviour of the developed inks, we measured viscosity as a function of shear rate to check for hysteresis and in a 3-step measurement.

Flow test comprises applying a pre-shear of 100 s-1 for 30 s followed by decreasing from 100 s-1 to 1 s-1 and subsequently increasing from 1 to 100 s-1. This test was repeated i) without pre-shear rate step, ii) 2 times with 5 min interval, and iii) starting from shear rate 1 s-1, increasing to 100 s-1, decreasing to 1 s-1. All cyclic results were identical and representative response of the inks was shown in *Fig. S2.* No hysteresis behaviour could be detected and ramp-up and ramp-down results are completely superimposed, suggesting the absence of time dependency during shear thinning (ramp-up) or structural construction (ramp-down) of polymer chains in the medium.





*Figure S2: Viscosity vs shear rate in a hysteresis representation*

In order support the time independency behaviour of inks, we applied 3-step test while fast data capturing (1000 points per second) was utilized to probe the viscosity as a function time during shear rate change. The 3-step test was applied as following; applying shear rate of 1 s^-1^ for 10 s, increasing the shear rate to 100 s^-1^ (i.e., deconstruction step) and dwell for 10 s, and then decrease to 1 s^-1^ (i.e., construction step) with dwell time of 10 s. Changing the shear rate in different steps took 0.2–0.5 s to reach the target. All tests were repeated i) 2 times and ii) after 5 min, showing identical results. Representative behaviour of developed PVP inks in construction-deconstruction steps is shown in *Fig. S3.*, demonstrating immediate viscosity change during shear rate adjustment. No time delay could be spotted and viscosity follows the shear rate instantaneously. Arrows show the time when motor adjusts the shear rate for the first time to the target value. This is followed by further fine adjustment by motor, especially in construction step, to stabilize the shear rate in the following few seconds.







*Figure S3: Deconstruction (black) and construction (red) steps in 3-step measurement for PVP inks for a.) 10 wt% and b.) 25 wt%*

**Printing dimensions**

For a typical ink composition, *Fig. S4* below shows how the nozzle diameter corresponded to the height of the extruded filament on the substrate (glass). The wetting resulted in the filament width on substrates being ~1.5× the nozzle diameter.

**
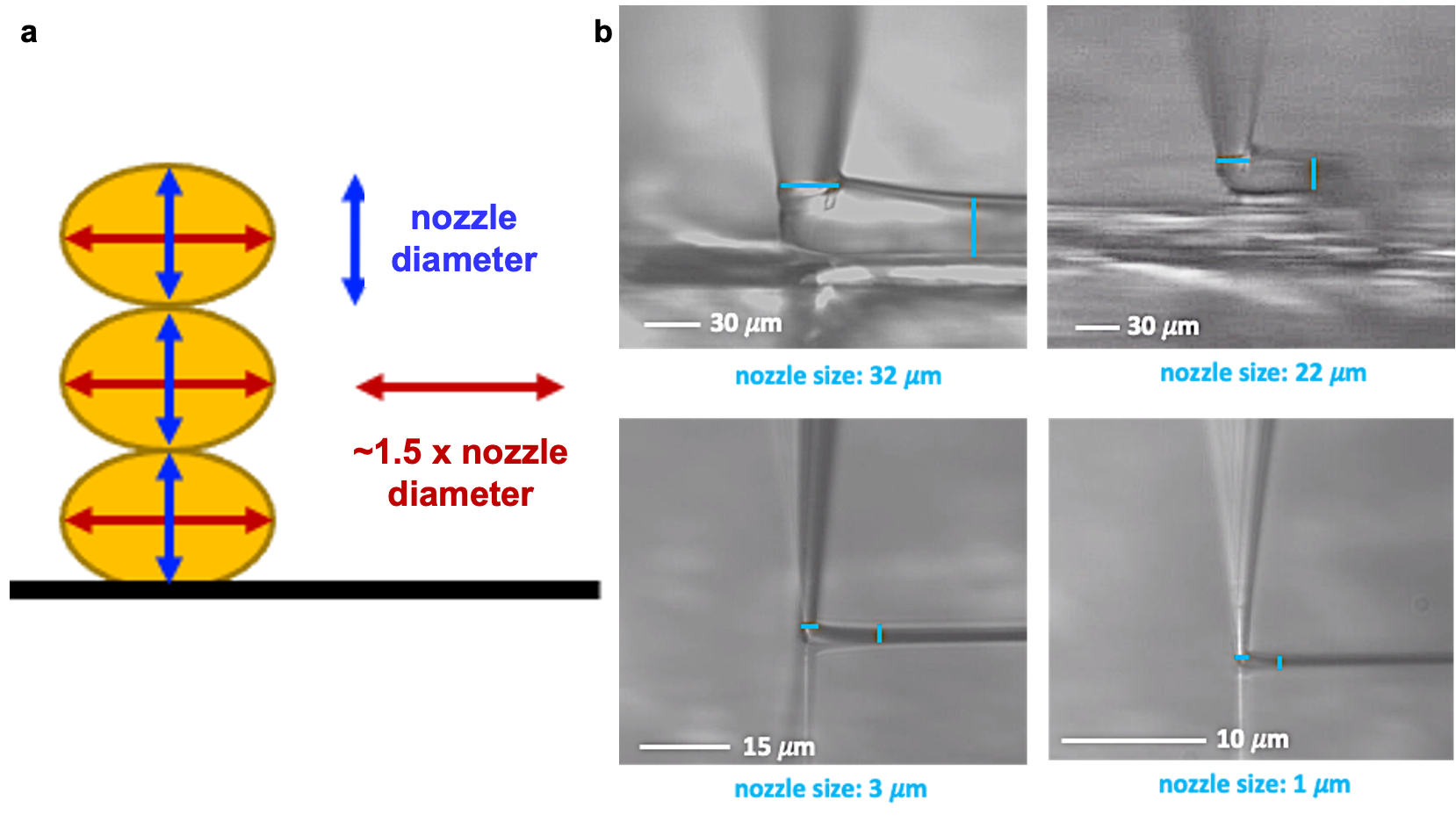
**

*Figure S4: a.) Schematic showing cross-section of extruded filaments with dimensions in relation to nozzle diameter b.) Measurements showing that the nozzle apertures and their extruded filament heights are approximately the same (Ink - 30 wt% PVP)*

**Quantitative phase diagram**

The phase diagram developed by Yuk and Zhao [1] maps different printing modes of viscoelastic inks with the help of non-dimensional parameters (H*, V*), where H*=H/αD and V*=V/C, with H being the gap between the tip and the substrate, α being the die-swelling ratio, D being the diameter of the tip, V being the tip moving speed and C being the extrusion rate. Our developed water based PVP inks all die-swell upon extrusion (V*≥1) and H*=0.41 (H=0.5*D, α=1.23) as shown in *Fig. S5a*. The phase diagram doesn’t cater for H*<1 as shown in *Fig. S5b*. Separately, the phase diagram doesn’t take into consideration mechanical failure of the nozzles, since the group uses metal tips that have apertures of ≥50 μm and inks that are solid-like (G’’/G’ < 1). But in our work we are working with much smaller nozzle apertures (1 to 35 μm) that are made of fragile glass capillaries that can break due to ink accumulation or clogging and our inks are liquid-like (G’’/G’ > 1). This is the reason why our plot is more suitable for our printing conditions, where mechanical failure of glass nozzles due to ink accumulation or clogging must be taken into account, since mechanical failure hinders printability.


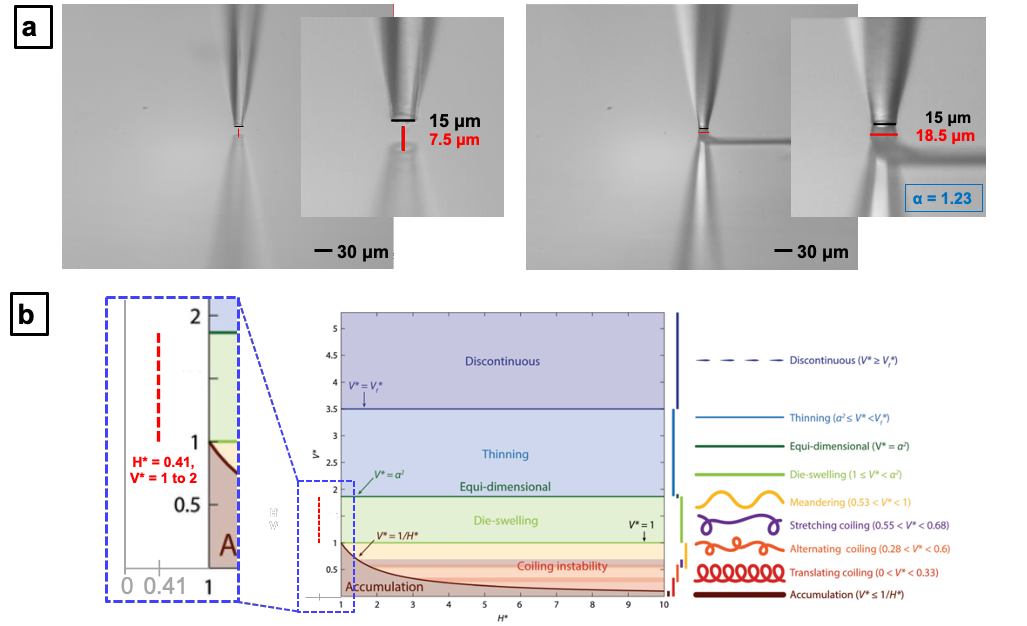


*Figure S5: a.) Measurements of H, D and determination of α b.) A quantitative phase diagram to guide the selection of printing modes (Discontinuous, Thinning, Equi-dimensional, Die-swelling, Coiling instability) for viscoelastic inks governed by nondimensional parameters (H*, V*)* [1]*. The dotted red line represents relevant region of our PVP ink and shows that it is outside the plot*

**References**

[1] H. Yuk, X. Zhao, A New 3D Printing Strategy by Harnessing Deformation, Instability, and Fracture of Viscoelastic Inks, Adv. Mater. 30 (2018). doi:10.1002/adma.201704028.
